# Food Fussiness, Fresh Fruit and Vegetable Consumption, and Their Correlations With Anthropometric Indices in Children

**DOI:** 10.1002/brb3.70336

**Published:** 2025-02-19

**Authors:** Nur Shahirah Mohd Tahir, Sharifah Intan Zainun Sharif Ishak, Seok Tyug Tan

**Affiliations:** ^1^ School of Graduate Studies Management and Science University Shah Alam Selangor Malaysia; ^2^ Department of Healthcare Professional, Faculty of Health and Life Sciences Management and Science University Shah Alam Selangor Malaysia; ^3^ Jeffrey Cheah School of Medicine and Health Sciences Monash University Malaysia Bandar Sunway Selangor Malaysia

**Keywords:** anthropometric indices, children, fresh fruit and vegetable consumption, fussy eating

## Abstract

**Introduction:**

Literature has consistently reported that fussy eaters usually have restricted food choices, particularly on fruits and vegetables. Insufficient intake of fruits and vegetables among fussy‐eating children can result in a lack of essential nutrients needed to support growth and development.

**Objective:**

This study aims to elucidate the relationships between food fussiness, fresh fruit and vegetable consumption, and anthropometric indices of children residing in Klang Valley, Malaysia.

**Methods:**

A cross‐sectional study was conducted to recruit 179 pairs of consenting caregiver‐child. Caregivers were required to report the surveyed child's sex, date of birth, and ethnicity. The six‐item food fussiness subscale from the Child Eating Behavior Questionnaire (CEBQ) was used to assess food fussiness in children. In addition, caregivers were asked to report whether their child had consumed fresh fruits and vegetables over the past month and to list all those they consistently refused to consume. For anthropometric measurements, children's body weight was measured with a digital bathroom scale, and height was measured using a portable stadiometer. Height‐for‐age *z*‐scores (HAZ) and BMI‐for‐age *z*‐scores (BAZ) were determined using the WHO Anthro software version 3.2.2 (for children below five) or the WHO AnthroPlus software version 1.0.4 (for children above five). The relationships between the studied variables were analyzed using IBM SPSS statistics version 27.0.

**Results:**

This study revealed that one in two children (54.2%) were fussy eaters, 9.5% did not consume fresh fruits, and 32.4% did not consume fresh vegetables over the past month. The findings from path analyses indicated that food fussiness was negatively correlated with fresh fruit and vegetable consumption. However, there were no significant direct and indirect relationships between food fussiness and anthropometric indices as indicated by HAZ and BAZ of children.

**Conclusion:**

The findings of this study demonstrated that food fussiness was negatively correlated with fresh fruit and vegetable consumption. Interventions can be carried out by encouraging children to consume fruits and vegetables they typically reject, such as bean vegetables, pear, papaya, and tuberous vegetables, to prevent nutrient deficiency.

## Graphical abstract

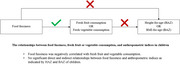

## 1 Introduction

Fussy eating (or picky eating) refers to rejecting or restricting familiar food and/or refusing to try unfamiliar food (or food neophobia) (Taylor et al. 2015). This atypical eating behavior usually peaks between the ages of 2 and 6 and persists throughout childhood (Cole et al. 2017; Taylor et al. 2015). The literature consistently reports that fussy eaters often have restricted food choices, which may lead to adverse health implications, including an increased risk of developing noncommunicable diseases such as type 2 diabetes, obesity, cardiovascular diseases, and certain cancers later in life (Estay et al. 2021; van der Horst et al. 2016). In addition, consuming a less varied diet among fussy eaters may also raise the risk of malnutrition and slower‐than‐expected growth. Evidence from two longitudinal studies indicates that fussy eaters are more likely to have lower body weight, fat‐free mass, and shorter stature compared to non‐fussy eaters (de Barse et al. 2015; Grulichova et al. 2022).

The Malaysian Dietary Guidelines for Children and Adolescents 2023 recommend that children aged 4–9 years should consume two servings of fruits and either two servings of vegetables (for children aged 4–6 years) or three servings of vegetables (for children aged 7–9 years) every day (National Coordinating Committee on Food and Nutrition 2023). However, the Southeast Asian Nutrition Survey (SEANUTS Malaysia) revealed that less than one‐fifth of children aged 1–6 years in Malaysia meet the daily recommended servings of fruits and vegetables as outlined in the Malaysian Dietary Guidelines for Children and Adolescents (Chong et al. 2017). It is well‐known that children require nutrients from diverse food sources for optimal growth. Fussy eating, which is often associated with the rejection of nutrient‐dense foods like fruits and vegetables (Fildes et al. 2016), can therefore be particularly concerning. As emerging evidence suggests that higher fruit and vegetable intake is negatively associated with BMI‐for‐age (Moffat et al. 2021) but positively associated with linear growth faltering (Parvin et al. 2022) in children, fussy eaters with insufficient fruit and vegetable intake may face a higher risk of obesity and shorter stature in the long run compared to non‐fussy eaters.

Although two studies have previously investigated the relationship between fussy eating and anthropometric indices in Malaysian children, these studies were centered around a narrow age range (preschoolers aged 5–6 years and primary school children aged 7–9 years) (Hanapi et al. 2022; Mok, Tung, and Kaur 2022). Although findings from the large‐scale UK Avon Longitudinal Study of Parents and Children (ALSPAC) indicated that fussy eaters strongly dislike fruits and vegetables (Taylor, Hays, and Emmett 2019), such a relationship has yet to be confirmed among children residing in Malaysia. In addition, the impacts of fussy eating on anthropometric indices, especially among those who reject fruits and vegetables, have remained understudied. Therefore, this study aims to elucidate the relationships between food fussiness, fresh fruit and vegetable consumption, and the anthropometric indices of children aged 3–8 years residing in Klang Valley, Malaysia.

## 2 Methodology

### 2.1 Study Design and Population

This cross‐sectional study was conducted from April 1st 2023 to June 30th 2023. Convenience and purposive sampling were adopted to recruit caregiver‐child pairs in the Klang Valley, Malaysia. Physically and mentally healthy children aged 3‐8 years who were free from clinically diagnosed eating disorders, along with their mentally healthy caregivers were included in this study. The prospective child‐caregiver pairs were recruited through convenience sampling from kindergartens and primary schools located in Klang Valley, Malaysia. Subsequently, purposive sampling was employed to ensure that the recruited child‐caregiver pairs met the specific inclusion criteria for this study.

The sample size was quantified using the G*Power software (version 3.1) with the application of a medium effect size (*f* ^2^ = 0.15) (Cohen 1988), a significance level (*α*) of 0.05, and a desired statistical power of 0.95. After accounting for a 20% dropout rate, this study would require recruiting a minimum of 143 caregiver‐child pairs.

### 2.2 Socio‐Demographic Characteristics and Anthropometric Measurements of Children

Caregivers were required to report the surveyed child's sex, date of birth, and ethnicity. Children's body weight was measured using the TANITA glass digital bathroom scale (HD‐378) and reported to the nearest 0.1 kg. Meanwhile, body height was quantified using the SECA portable stadiometer (SECA 213) and reported to the nearest 0.1 cm. Height‐for‐age *z*‐scores (HAZ) and BMI‐for‐age *z*‐scores (BAZ) were determined using the WHO Anthro software version 3.2.2 (for children below five) (World Health Organization 2010) or the WHO AnthroPlus software version 1.0.4 (for children above five) (World Health Organization 2009). The *z*‐scores of the abovementioned growth indicators were interpreted according to the recommendations of the World Health Organization (2006).

### 2.3 Food Fussiness Among Children

The six‐item food fussiness subscale from the Child Eating Behavior Questionnaire (CEBQ) was used to assess food fussiness in children (Wardle et al. 2001). All items were on a five‐point Likert scale ranging from “never” to “always.” Scoring for normal score items (three‐item) was based on a scale from 1 to 5, with 1 = “never,” 2 = “rarely,” 3 = “sometimes,” 4 = “often,” and 5 = “always.” In contrast, the scoring was reversed for the reverse scoring items (three‐item). Responses from the six‐item were then averaged for the mean score. Children were classified into two groups based on their mean score in the food fussiness subscale: non‐fussy eaters (<3.00) and fussy eaters (≥3.00) (Steinsbekk et al. 2017). The reliability of the food fussiness subscale was acceptable (Cronbach's alpha = 0.747).

### 2.4 Fresh Fruit and Vegetable Consumption

Caregivers were asked to respond to the following questions using binary responses (Yes/No): “Has your child consumed fresh fruits over the past month?” and “Has your child consumed fresh vegetables over the past month?” Caregivers who responded negatively to one or both questions were required to list all fresh fruits and vegetables that the surveyed child consistently refused to consume.

### 2.5 Statistical Analysis

Data were analyzed using IBM SPSS statistics version 27.0 (IBM Corp, Armonk, NY). Descriptive statistics (frequency, percentage, mean, and standard deviation) were used to describe variables when appropriate. The associations between food fussiness and fresh fruit and vegetable consumption over the past month were analyzed using Pearson's chi‐square test. Socio‐demographic covariates that could potentially affect the relationships between the studied variables were identified using Pearson's correlation test. Socio‐demographics that portray a *p* value of less than 0.15 (*p* < 0.15) in relation to food fussiness, fresh fruit and vegetable consumption or anthropometric indices (HAZ and BAZ) were subsequently selected as covariates for the path analyses. Two path analyses with the adjustment for the age and ethnicity of the children were conducted to examine the relationships between food fussiness, fresh fruit and vegetable consumption, and anthropometric indices (HAZ and BAZ) (Figure 2a,b). These analyses were performed using Model 4 of the PROCESS macro for SPSS (Hayes 2022), with 5000 bootstrap resamples at a 95% confidence level. Statistical significance was set at a *p* value of less than 0.05 (*p *< 0.05).

## 3 Results

Table 1 shows the socio‐demographics and anthropometric indices of the surveyed children. This study was predominantly composed of girls (*n* = 98, 54.7%), children aged 3–5 years (*n* = 99, 55.3%), and Malays (*n* = 129, 72.1%). Although slightly more than four‐fifths (*n* = 144, 80.4%) had normal HAZ, close to half were either severely thin or thin (*n* = 89, 49.7%), as indicated by BAZ.

**TABLE 1:** Socio‐demographics and anthropometric indices of the surveyed children.

Socio‐demographics	Frequency, *n* (%)	Mean ± standard deviation
**Sex**		
Boy	81 (45.3)	
Girl	98 (54.7)	—
**Age (years old)**		
3–5	99 (55.3)	
6–8	80 (44.7)	5.83** ± **0.92
**Ethnicity**		
Malay	129 (72.1)	
Chinese	43 (24.0)	—
Indian	7 (3.9)	
**Height‐for‐age *z*‐scores (HAZ)**		
Severely stunting/stunting	10 (5.6)	
Normal	144 (80.4)	0.95** ± **1.80
Very tall	25 (14.0)	
**BMI‐for‐age *z*‐scores (BAZ)**		
Severely thinness/thinness	89 (49.7)	
Normal	81 (45.3)	−2.06** ± **1.96
Overweight/obese	9 (5.0)	

Table 2 indicates the prevalence of food fussiness and fresh fruit and vegetable consumption over the past month. Of the 179, it is observed that 1 in 2 (*n* = 97, 54.2%) were fussy eaters, 9.5% (*n* = 17) did not consume fresh fruits, and approximately one‐third (*n* = 58, 32.4%) did not consume fresh vegetables over the past month. Additional analysis was carried out to examine fresh fruit and vegetable consumption over the past month among non‐fussy eaters and fussy eaters (Table 3). Of the 82 non‐fussy eaters, 5 (6.1%) did not consume any fresh fruits, and 19 (23.2%) did not consume any fresh vegetables. The proportion of those who did not consume fresh fruits and vegetables among fussy eaters (*n* = 97) is even higher, with 39 (40.2%) not consuming fresh vegetables and 12 (12.4%) not consuming fresh fruits. Interestingly, the findings from Pearson's chi‐square test indicated that food fussiness was associated with fresh vegetable consumption (*χ* ^2^ = 5.888, *p *= 0.015).

**TABLE 2:** The prevalence of food fussiness and fresh fruit and vegetable consumption over the past month.

Indicators	Frequency, *n* (%)
**Food fussiness**	
Non‐fussy eaters	82 (45.8)
Fussy eaters	97 (54.2)
**Has your child consumed fresh fruit over the past month?**	
No	17 (9.5)
Yes	162 (90.5)
**Has your child consumed fresh vegetables over the past month?**	
No	58 (32.4)
Yes	121 (67.6)

**TABLE 3:** Fresh fruit and vegetable consumption over the past month among non‐fussy eaters and fussy eaters.

Fresh fruit and vegetable consumption over the past month	Frequency, *n* (%)		*χ* ^2^ (*p* value)
	Non‐fussy eaters (*n* = 82)	Fussy eaters (*n* = 97)	
Did not consume any fresh fruit	5 (6.1)	12 (12.4)	2.035 (0.154)
Consumed at least one fresh fruit	77 (93.9)	85 (87.6)	
Did not consume any fresh vegetable	19 (23.2)	39 (40.2)	5.888 (0.015)*
Consumed at least one fresh vegetable	63 (76.8)	58 (59.8)	

*Statistical significance was considered at *p *< 0.05.

**FIGURE 1:**
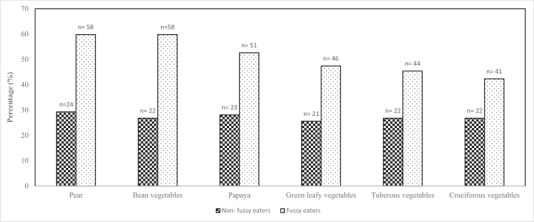
Fresh fruits and vegetables rejected by non‐fussy eaters and fussy eaters. (1). Caregivers were allowed to list more than one fruit and vegetable. (2) Examples of bean vegetables: long beans, winged beans, lady's finger (okra) and bean sprouts. (3) Examples of green leafy vegetables: mustard greens (*sawi*), spinach (*bayam*), water spinach (*kangkung*), and Chinese broccoli (*kailan*). (4) Examples of tuberous vegetables: potato, pumpkin, maise, and taro. (5) Examples of cruciferous vegetables: cabbage, broccoli, and cauliflower.

Figure 1 shows the fresh fruits and vegetables rejected by non‐fussy eaters and fussy eaters. It is observed that pear (*n* = 24, 29.3%), papaya (*n* = 23, 28.1%), bean vegetables (*n* = 22, 26.8%), tuberous vegetables (*n* = 22, 26.8%), and cruciferous vegetables (*n* = 22, 26.8%) were the top five fresh fruits and vegetables rejected by non‐fussy eaters. The types of fresh fruits and vegetables rejected by fussy eaters are very similar to those rejected by non‐fussy eaters, except that the number of rejections for each listed fruit and vegetable is even higher among fussy eaters than non‐fussy eaters. Among the fussy eaters, the top five most rejected fruits and vegetables were pear (*n* = 58, 59.8%), bean vegetables (*n* = 58, 59.8%), papaya (*n* = 51, 52.6%), green leafy vegetables (*n* = 46, 47.4%), and tuberous vegetables (*n* = 44, 45.4%).

**FIGURE 2:**
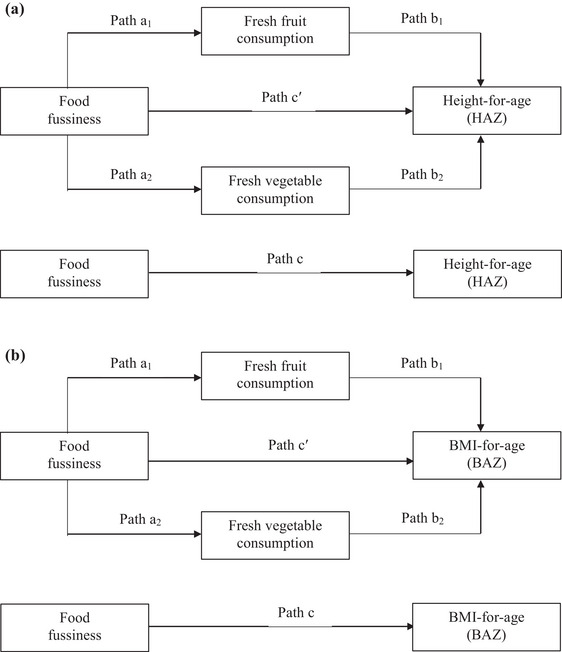
(a) Statistical diagram illustrating the path analysis of the relationships between food fussiness, fresh fruit and vegetable consumption, and height‐for‐age (HAZ). (b) Statistical diagram illustrating the path analysis of the relationships between food fussiness, fresh fruit and vegetable consumption, and BMI‐for‐age (BAZ).

Age and ethnicity were selected as covariates in the subsequent path analyses because these variables showed a *p* value of less than 0.15 with fresh vegetable consumption (ethnicity: *r* = −0.138, *p *= 0.065), HAZ (age: *r *= ‐0.110, *p *= 0.144 and ethnicity: *r *= 0.256, *p *< 0.001) and BAZ (age: *r *= 0.189, *p *= 0.011 and ethnicity: *r *= ‐0.213, *p *= 0.004) (Table 4). Two path analyses with the adjustment for age and ethnicity were conducted in order to examine the relationships between food fussiness, fresh fruit and vegetable consumption, and anthropometric indices (Figure 2a,b). Although food fussiness was significantly and negatively correlated with fresh fruit consumption (*B *= −0.099, SE = 0.003, *p *< 0.001) and fresh vegetable consumption (*B *= −0.203, SE = 0.003, *p *< 0.001), no significant correlations were observed between fresh fruit consumption with HAZ (*B *= 1.351, SE = 7.910, *p *= 0.865) and BAZ (B = 4.726, SE = 8.597, *p *= 0.583) as well as between fresh vegetable consumption with HAZ (B = 4.888, SE = 7.830, *p *= 0.533) and BAZ (B = −10.92, SE = 8.510, *p *= 0.201). The indirect and direct relationships between food fussiness and anthropometric indices (HAZ and BAZ) were also investigated in this study. However, these relationships were reported to be insignificant (Table 5).

**TABLE 4:** Correlations between socio‐demographics, fussy eating, fresh fruit and vegetable consumption, and anthropometric indices of children.

Socio‐demographics	Fresh fruit consumption	Fresh vegetable consumption	Food fussiness	HAZ	BAZ
**Sex**	0.088 (0.240)	0.066 (0.380)	0.017 (0.819)	−0.023 (0.762)	−0.012 (0.869)
**Age**	0.003 (0.971)	0.076 (0.310)	−0.061 (0.419)	−**0.110 (0.144)**	**0.189 (0.011)**
**Ethnicity**	−0.032 (0.673)	−**0.138 (0.065)**	−0.004 (0.960)	**0.256 (<0.001)**	−**0.213 (0.004)**

*Note*: Socio‐demographics with a *p* value of less than 0.15 (*p *< 0.15) were selected as covariates in the path analyses.

Abbreviations: BAZ, BMI‐for‐age *z*‐scores; HAZ, Height‐for‐age *z*‐scores.

**TABLE 5:** The path analyses of the relationships between food fussiness, fresh fruit and vegetable consumption, and anthropometric indices of children.

Path^a^	B	SE	*t* value	*p* value	LLCI	ULCI
**Path a_1_**						
Food fussiness → Fresh fruit consumption	−0.099	0.003	−30.61	<0.001*	−0.106	−0.093
**Path a_2_**						
Food fussiness → Fresh vegetable consumption	−0.203	0.003	−62.07	<0.001*	−0.210	−0.197
**Path b_1_**						
Fresh fruit consumption → HAZ	1.351	7.910	0.017	0.865	−14.26	16.96
Fresh fruit consumption → BAZ	4.726	8.597	0.550	0.583	−12.24	21.70
**Path b_2_**						
Fresh vegetable consumption → HAZ	4.888	7.830	0.624	0.533	−10.57	20.34
Fresh vegetable consumption → BAZ	−10.92	8.510	−1.283	0.201	−27.72	5.879
**Path c′**						
Food fussiness → HAZ	0.984	1.091	0.902	0.368	−1.169	3.137
Food fussiness → BAZ	−1.948	1.186	−1.643	0.102	−4.288	0.392
**Path c**						
Food fussiness → HAZ	−0.144	0.203	−0.710	0.478	−0.545	0.256
Food fussiness → BAZ	−0.197	0.221	−0.890	0.375	−0.633	0.240

*Note*: Height‐for‐age (HAZ), BMI‐for‐age (BAZ), B = unstandardised coefficient, SE = Standard error, LLCI = Lower limit confidence interval and ULCI = Upper limit confidence interval.

Abbreviations: BAZ, BMI‐for‐age *z*‐scores; HAZ, height‐for‐age *z*‐scores.

^a^ Path analyses were conducted with adjustments for age and ethnicity.

*Statistical significance was considered at *p *< 0.05.

## 4 Discussion

Two local studies delineated that the prevalence of fussy eating (picky eating) was 31.8% among preschoolers (5–6 years old) (Hanapi et al. 2022) and 38.0% among school‐age children (7–9 years old) (Mok, Tung, and Kaur 2022). This study observed that 54.2% of children aged 3–8 years were fussy eaters, indicating that fussy eating is 1.4–1.7 times more prevalent than those reported in the literature. The exceptionally high prevalence in this study may be attributed to a broader age range covered compared to the literature. According to the latest National Health and Morbidity Survey 2022 (NHMS 2022), 21.2% of children under five in Malaysia are stunted, 15.3% are underweight, and 5.6% are overweight (Institute for Public Health 2023). Even though the proportions of stunted (5.6%) and overweight/obese (5.0%) children in the current study are lower compared to those reported in NHMS (2022), the proportion of children with thinness/severe thinness in this study is slightly more than double (49.7%) (Table 1). The significant difference in body weight status could be attributed to this study being conducted in a single geographical area (Klang Valley) and having a smaller sample size compared to the nationwide NHMS survey.

To the best of the authors’ knowledge, no prior studies have reported the proportion of children in Malaysia who do not consume fresh fruits and vegetables at all. However, the findings showed that 9.5% of children did not consume fresh fruits, and 32.4% did not consume fresh vegetables over the past month (Table 2). This suggests that children are more likely to avoid vegetables, which often have a bitter taste, compared to fruits, which typically have a sweet taste (Mashitah, Nur Azreen, and Zahara 2020). Although it has been previously reported that all children (regardless of their fussy eating status) have a higher tendency to reject fruits and vegetables (Kamarudin et al. 2023), the findings of this study demonstrated that fussy eaters were more likely to reject fresh vegetables than non‐fussy eaters (Table 3). It is also worth noting that children in Malaysia (regardless of their fussy eating status) showed the least preference for bean vegetables, tuberous vegetables, pear, and papaya. In general, these findings also align with a recent study conducted by Mashitah, Nur Azreen, and Zahara (2020), which indicates that Malay children aged 9–12 years had the least preference for fresh fruits such as kiwi, guava, pear, papaya, and pineapple, as well as fresh vegetables such as string beans, kale, and cauliflower. Children may develop a strong taste and texture aversion toward the listed fresh fruits and vegetables in this study due to their fibrous, soft, mushy textures, bitter taste, or appearance (Chow et al. 2024).

Another interesting finding worth highlighting is that ethnicity was positively correlated with HAZ but negatively correlated with BAZ (Table 4). This could be attributed to differences in dietary practices among various ethnic groups in Malaysia, as well as variations in familial socio‐economic status (Jeinie et al. 2021). It is observed that food fussiness was negatively correlated with fresh fruit and vegetable consumption after adjusting for the age and ethnicity of children (Table 5). Overall, these findings are consistent with a recent study conducted in three European countries (Italy, Poland, and the United Kingdom), which suggests that fussy eaters consumed fewer fruits and vegetables compared to non‐fussy eaters (Masento et al. 2022). In addition to taste and texture aversion, lack of early exposure to fruits and vegetables (Mallan et al. 2016), parental feeding practices (such as a lack of encouragement and parental modeling to consume fruits and vegetables) (Harmancioǧlu and Kabaran 2023), and the availability of fruits and vegetables at home (Harris et al. 2019) are also among the factors contributing to lower fruit and vegetable consumption among fussy eaters.

Although several studies have investigated the association between food fussiness and anthropometric indices of children, the findings have remained conflicting and ambiguous. Although a 15‐year European Longitudinal Study of Pregnancy and Childhood (ELSPAC–CZ) showed that fussy eaters were on average 2.3 kg lighter and 0.8 cm shorter than non‐fussy eaters (Grulichova et al. 2022), a cross‐sectional study conducted in the United States failed to observe an association between food fussiness and BMI‐for‐age among preschoolers (Brown et al. 2018). Coincidentally, the findings of this study also demonstrated that there were no significant correlations between food fussiness and anthropometric indices (HAZ and BAZ). In addition, the findings of this study suggested that fresh fruit and vegetable consumption did not mediate the relationships between food fussiness and anthropometric indices (Table 5). The lack of association could be attributed to the fact that overall calorie intake, rather than food fussiness or fruit and vegetable consumption patterns, is the primary determinant of body weight status among children (American Heart Association 2024; Kamarudin et al. 2023). Given the multifaceted nature of factors affecting children's growth, it is also important to consider household socio‐economic status (such as household income, parental education level, and employment status), household food security status, lifestyle behavior of the child (such as physical activity level), parental feeding beliefs, and practices when evaluating anthropometric indices of children (Chong et al. 2017; Cole et al. 2017; Sharmilla and Tan 2022).

The findings of this study should be interpreted within the context of its limitations. First, this study only investigated the relationships between food fussiness, fresh fruit and vegetable consumption, and anthropometric indices of children aged 3–8 years. To provide a more comprehensive justification for the relationship between food fussiness and the weight status of fussy eaters, future studies may consider assessing overall dietary patterns using the food frequency questionnaire (FFQ) and including children of all ages. Second, this study did not examine seasonal variation in fruit and vegetable intakes among children. Third, the low mean BMI‐for‐age in this study (BAZ = −2.06 ± 1.96) may not represent the general child population, which typically has an average BAZ closer to 0. Fourth, data collection for this cross‐sectional study was carried out in the Klang Valley of Malaysia. Therefore, the findings from this study might be impossible to generalize to all children residing in Malaysia.

## 5 Conclusion

In a nutshell, the findings of this study demonstrated that food fussiness was negatively correlated with fresh fruit and vegetable consumption. However, there were no significant direct and indirect relationships between food fussiness and anthropometric indices as indicated by HAZ and BAZ of children. Interventions can be carried out by encouraging children to consume fruits and vegetables they typically reject, such as bean vegetables, pear, papaya, and tuberous vegetables, to prevent nutrient deficiency.

## Author Contributions

All authors contributed to the conception, design, material preparation, data collection, and analysis. Nur Shahirah Mohd Tahir wrote the first draft of the manuscript, and all authors commented on previous versions. All authors read and approved the final manuscript.

## Ethics Statement

This study protocol was reviewed and approved by the Research Ethics Committee of Management and Science University, approval number MSU‐RMC‐02/FR01/01/L1/031.

## Consent

Informed consent was obtained from the parents or caregivers before data collection.

## Conflicts of Interest

The authors declare no conflicts of interest.

### Peer Review

The peer review history for this article is available at https://publons.com/publon/10.1002/brb3.70336.

## Data Availability

The datasets generated during and/or analyzed during the current study are available from the corresponding author upon reasonable request.
